# Epidemiology of Pelvic Floor Dysfunction in the Tacna Region of Peru, 2023

**DOI:** 10.1007/s00192-024-05792-6

**Published:** 2024-05-09

**Authors:** Gema Sologuren-García, Carmen L. Linares, Jackeline R. Flores, Gloria Escobar-Bermejo, Soledad Sotelo-Gonzales, Cristhel K. Fagerstrom

**Affiliations:** 1 Universidad Nacional Jorge Basadre Grohmann Urb, Santa Ana A-22, Tacna, Peru; 2 Escuela de Obstetricia y Puericultura, Facultad de Medicina, Universidad Andres Bello, 8370134 Santiago, Chile

**Keywords:** Pelvic floor dysfunction, Quality of life, Urinary incontinence

## Abstract

**Introduction and hypothesis:**

Urinary incontinence (UI) is highly prevalent in the general population, with numerous studies conducted in Europe and North America. However, there is a scarcity of data regarding its prevalence and sociodemographic factors in the southern region of Peru. There is an association between sociodemographic factors—such as age, educational level, body mass index, number of pregnancies, parity, mode of delivery, weight of the newborn—along with lifestyle factors such as physical effort, coffee and tobacco consumption with pelvic floor dysfunction (PFD). We anticipate that this association will negatively impact women’s quality of life.

**Methods:**

This was a quantitative study, with a non-experimental, descriptive, cross-sectional correlational design. A sample consisting of 468 women between 30 and 64 years old. A previously tested survey was applied to explore prevalence, symptoms, associated factors, and quality of life.

**Results:**

The prevalence of PFD was 73.9%. UI was the most common. There is a significant association with overweight, obesity, parity, route of delivery, and physical effort. Even though a large percentage of participants presented with PFD, they reported that their quality of life was not affected (65.9% urinary discomfort, 96.5% colorectal–anal discomfort and 92.2% pelvic organ prolapse discomfort) and only in the case of urinary discomfort did they state that the impact was mild (28.6%) and moderate (5.5%).

**Conclusions:**

Pelvic floor dysfunction in women is very common and it is strongly associated with overweight, obesity, parity, route of delivery, and physical exertion. The impact on quality of life was mild and moderate for those who had urinary discomfort.

## Introduction

The pelvic floor comprises muscles, ligaments, and fascia; this integration is essential for the stability and muscle tone of the pelvic girdle, continence, urination/defecation, and sexuality, among others.

Pelvic floor dysfunction (PFD) in women encompasses a wide range of clinical disorders: urinary incontinence (UI), pelvic organ prolapse (POP), fecal incontinence (FI), and pain syndrome of the pelvic–perineal region. It is a prevalent and underdiagnosed problem worldwide [1].

If broken down by frequency, the most common disorders are urinary incontinence, followed by POP and FI. Finding one of these dysfunctions in non-institutionalized women is at least 23% [2]; percentages range from 10 to 50% depending on the age and number of births of the group surveyed.

A large number of surveys and studies carried out in the last 20 years have led to greater clarity about the risk factors for these pathological conditions, highlighting age, number of pregnancies, mode of delivery, obesity, and lifestyle [3].

### Urinary Incontinence and Quality of Life

Recent years have seen a general increase in studies on the prevalence of UI, as well as its associations with quality of life in different contexts (economic, social, health, etc.).

There are several validated instruments for the assessment of quality of life, such as "The Short Form-36 Health Survey (SF-36)," which addresses self-perception of health, activities of daily living, work, social activities, depressive symptoms, and quality of life, among others. This instrument is used in more than 40 countries and has been modified for application in older adults in the USA, the UK, and Australia [4, 5]. In Spain, the SF-36 showed good discrimination between severity groups and moderate correlation with clinical indicators, which is fundamental as it has been validated in Spanish [6].

A study in Portugal associated UI with quality of life assessed by means of the SF-36 questionnaire, carried out in four large hospital centers, where users with UI (*N* = 505) were surveyed. A negative impact on quality of life was found in 501 of the participants (99.2%). This impact was classified as mild, moderate, severe, and very severe, with 72.5% of the population aged 50 years and over falling into the latter two categories. Furthermore, compared with younger participants, women in this age group had more sleep disturbances and energy and performance limitations (*p* ≤ 0.04) compared with those under 50 years of age [7].

### Obstetricians and Pelvic Floor Dysfunction

Obstetric professionals, who have a 5-year university education, are trained to provide health care for women and families throughout the life cycle. In this context, they are usually the first professional to consult in pathological conditions of the urogenital sphere and are qualified to provide education, prevention, treatment, and initial and nonsurgical management of pelviperineal pathological conditions, as they are low-risk and minimally invasive interventions [8], which lead to rapid improvement of patients in most cases [9].

According to the data previously presented, there are millions of people in the world who suffer from PFD, with alterations in their quality of life that can be severe and associated with millions in health care costs, both for hospital services and for their own economies [10]; therefore, it is essential to know in our community the prevalence, distribution of dysfunction, and self-perception of the decrease in quality of life associated with it, in order to begin working on effective therapeutic measures and improvement plans.

Therefore, the aim of the study was to determine the association of socio-demographic factors such as age, educational level, body mass index, number of pregnancies, parity, delivery route, birth weight, physical exertion, and coffee and tobacco consumption with PFD, and to establish their impact on women's quality of life.

### Methodology, Design and Study Population

This was a quantitative, non-experimental, descriptive, correlational, and cross-sectional study design. The population consisted of 70,924 women aged 30 to 64 years, assigned to the health network of the Ministry of Health of the Tacna region of Peru in 2023.

The sample consisted of 468 women, stratified by micro-networks and randomly selected health facilities. All women who agreed to participate voluntarily and signed the informed consent form were included.

Ethical approval for the study was obtained from the Institutional Research Ethics Committee of the Hospital, assigning it the code: CIÉI-HHUT: 37-CIÉI-2023.

### Variables and Dimensions

There were three study variables: 1) Sociodemographic (age, educational level, occupation, marital status, has current partner, religion, origin, body mass index, obstetric formula, route of delivery, heaviest newborn, performs physical exertion, chronic cough, smoking, coffee consumption, amount of coffee, alcohol consumption), 2) Pelvic floor discomfort (urinary discomfort, colorectal–anal discomfort and pelvic organ prolapse discomfort) and 3) Impact of pelvic floor on quality of life.

### Technique, Instrument, and Data Processing

The survey technique was used to collect data from two instruments to determine the prevalence and socio-demographic parameters associated with PFD and its impact on women's quality of life. The Pelvic Floor Discomfort Inventory (PFDI-20), in its short version, was used, which assesses discomfort related to pelvic floor symptoms and includes three scales: Urinary Discomfort Inventory (UDI-6), Pelvic Organ Prolapse Discomfort Inventory (POPDI-6), and Colorectal–Anal Discomfort Inventory (CRADI-8), each with their respective scores. The score is obtained by averaging the sum and number of items and finally multiplying by 25. The total is obtained by summing the three scales: the higher the values, the greater the discomfort. The values of the total score classify the degree of discomfort: mild (1–15), moderate (16–34), and severe (35–40) [11, 12].

The Pelvic Floor Impact Questionnaire (PFIQ-7), in its short version, was also used, which assesses the impact of pelvic floor dysfunction on the lives of women with pelvic floor dysfunction. It consists of seven questions to be answered three times each (corresponding to no, mild, moderate, and severe) considering symptoms related to bladder or urine, vagina or pelvis, and bowel or rectum, and their effect on function, social health, and mental health in the last 3 months. Responses to each question range from "not at all" (0) to "quite a lot" (3). To obtain scale scores, the mean of each of the 3 is calculated individually, ranging from 0 to 3; this number is then multiplied by 100 and then divided by 3. The scale scores are then summed to obtain the total score of the PFIQ-7, which ranges from 0 to 300. A lower score means that there is a lower effect on quality of life [12].

Several studies corroborate both questionnaires having been proven to be psychometrically valid and reliable. Both instruments were adapted to short versions in 2005 and validated in other languages such as French, Swedish, Chinese, Arabic, Turkish, and, recently, Spanish by Spanish speakers in the USA [13]. The present study used the validated Spanish versions of every survey.

### Procedure and Statistical Analysis

It was carried out using SPSS version 27 statistical software and Excel spreadsheet. The data were presented using one- and two-dimensional frequency tables.

The existence of association was assessed using Kendall's Tau-c statistic, Fisher's exact test, and Chi-squared test, with a confidence level of 95%, a proportion of 0.5, and an estimation error of 0.045.

## Results

**Fig. 1:**
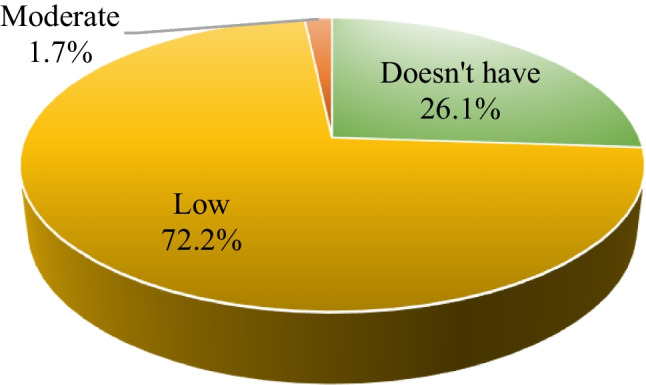
Level of dysfunction of the pelvic floor

The prevalence of PFD in women aged 30–64 years was 73.9% (346 out of 468; (Fig. 1).

Fifty percent and 43.8% had mild and moderate PFD respectively, with ages ranging from 30 to 39 years and educational level ranging from secondary to higher education (Table 1). With a significance level of α = 0.005, an association was found between: body mass index, number of gestations, total number of deliveries, and number of vaginal deliveries with the level of PFD. There was no association between harmful habits and PFD (Tables 2, 3).

**Table 1:** Sociodemographic variables according to the level of pelvic floor dysfunction

Variable	Level of dysfunction					
	Low		Moderate		Total	
	*N*°	%	*N*°	%	*N*°	%
Age						
30 to 39	148	43.8	4	50	152	43.9
40 to 49	91	26.9	2	25	93	26.9
50 to 59	71	21	1	12.5	72	20.8
60 to 69	28	8.3	1	12.5	29	8.4
Educational level						
Without	8	2.4	0	0	8	2.3
Primary education	63	18.6	1	12.5	64	18.5
Secondary education	166	49.1	4	50	170	49.1
Higher Education	101	29.9	3	37.5	104	30.1
Body mass index						
Normal	73	21.6	0	0	73	21.1
Overweight	133	39.3	2	25	135	39
Obesity	107	31.7	6	75	113	32.7
Not specified	25	7.4	0	0	25	7.2
Number of pregnancies						
0	31	9.2	0	0	31	8.9
1	56	16.6	0	0	56	16.2
2 a +	251	74.3	8	100	259	74.9
Parity						
Nullipara	4	1.3	0	0	4	1.3
Primipara	74	24.1	0	0	74	23.5
Multipara	229	74.6	8	100	237	75.2
Mode of delivery						
Vaginal	199	65.7	7	87.5	206	66.2
Cesarean	60	19.8	1	12.5	61	19.6
Vaginal and cesarean	44	14.5	0	0	44	14.1
Newborn weight						
Appropriate weight	225	74.3	7	87.5	232	74.6
Macrosomic	72	23.8	2	2.5	73	23.5
Not specified	6	2	0	0	6	1.9
Physical exertion						
No	220	65.1	6	75	226	65.3
Yes	118	34.9	2	25	120	34.7
Type of exertion						
No exertion	220	65.1	6	75	226	65.3
Weightlifting	108	32	2	25	110	31.8
Not specified	10	3	0	0	2.9	
Chronic cough						
No	316	93.5	8	100	324	93.6
Yes	22	6.5	0	0	22	6.4

**Table 2:** Harmful habits according to the level of pelvic floor dysfunction

Variable	Level of dysfunction					
	Low		Moderate		Total	
	*N*°	%	*N*°	%	*N*°	%
Cigarette consumption						
No	335	99.1	8	100	343	99.1
Yes	3	0.9	0	0	3	0.9
Coffee consumption						
No	245	72.5	4	50	249	72
Yes	93	27.5	4	50	97	28
Amount of coffee						
1 cup a day (<200 mg)	79	84.9	3	75	82	81.4
From 2 to 4 cups a day (100 to 400 mg)	14	15.1	1	25	15	13.4
Alcohol consumption						
No	233	68.9	3	37.5	236	68.2
Yes	105	31.1	5	62.5	110	31.8

**Table 3:** Test indicators and statistics

Association		Statistical test	Significance
Age range	Level of pelvic floor dysfunction	Kendalls’s Tau-c	0.795
Body mass index	Level of pelvic floor dysfunction	Kendalls’s Tau-c	**0.032**
Quantity of pregnancies	Level of pelvic floor dysfunction	Kendalls’s Tau-c	**0.005**
Total number of deliveries	Level of pelvic floor dysfunction	Kendalls’s Tau-c	**0.005**
Number of vaginal deliveries	Level of pelvic floor dysfunction	Kendalls’s Tau-c	**0.008**
Has chronic cough or not	Level of pelvic floor dysfunction	Fisher’s exact test	0.588
Consumes cigarettes or not	Level of pelvic floor dysfunction	Fisher’s exact test	0.932
Consumes coffee or not	Level of pelvic floor dysfunction	Fisher’s exact test	0.157
Quantity of coffee consumed	Level of pelvic floor dysfunction	Kendalls’s Tau-c	0.658
Alcohol consumption	Level of pelvic floor dysfunction	Fisher’s exact test	0.071

Significance level: α = 0.005; entries in bold are significant

**Fig. 2:**
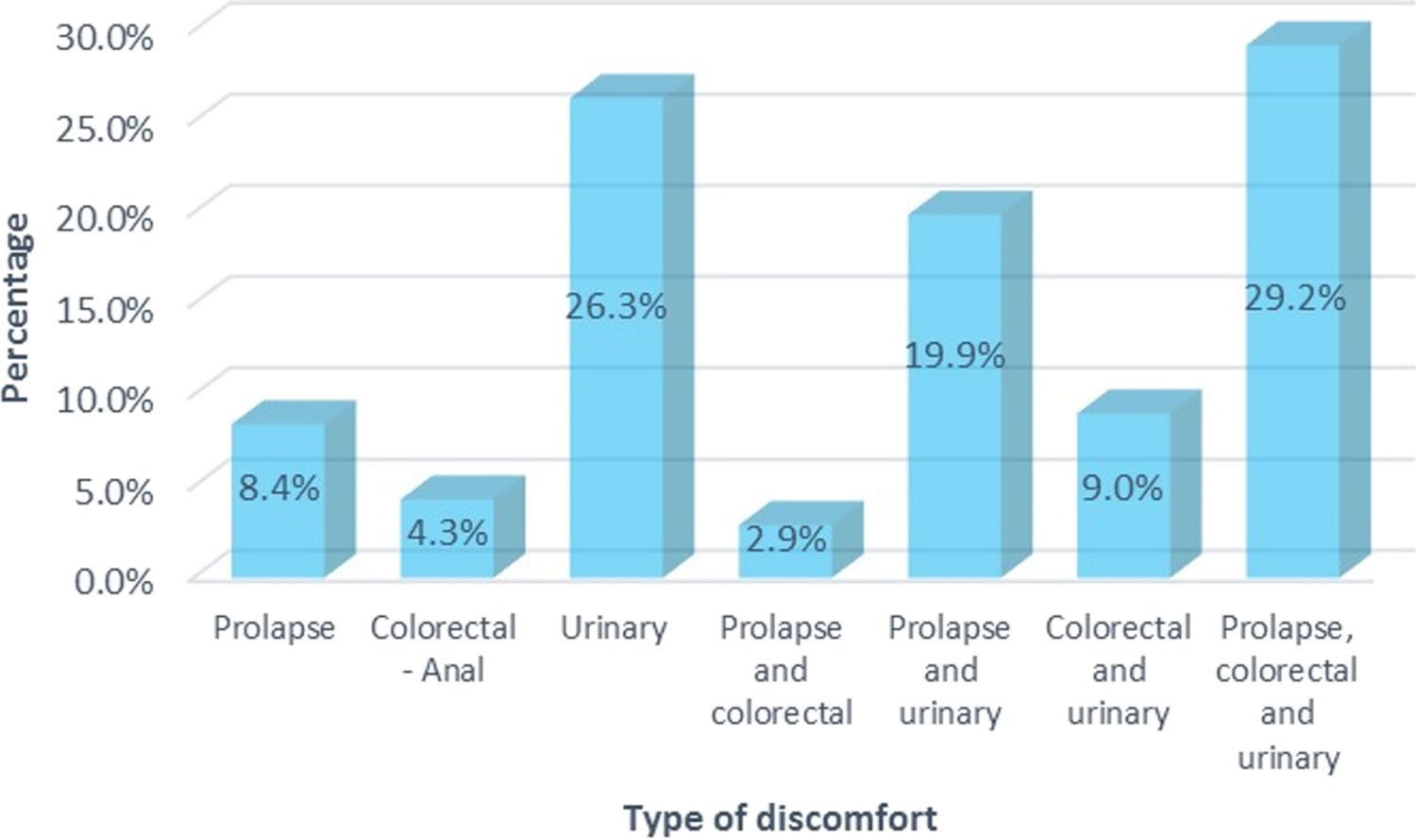
Reported symptomatology

Figure 2 shows that 29.2% present all three types of discomfort (pelvic organ prolapse, colorectal and urinary discomfort), only 26.3% report urinary discomfort, and 19.9% have discomfort due to prolapse and urinary discomfort.

**Fig. 3:**
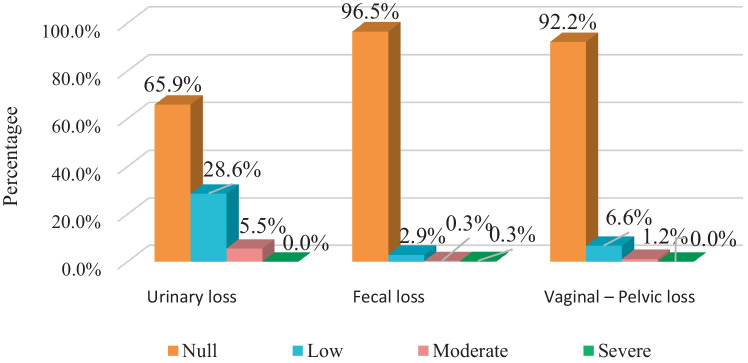
Pelvic floor dysfunction and its impact on quality of life

It is striking that in spite of presenting the three types of discomfort (urinary, colorectal, and pelvic organ prolapse), the majority reported that their quality of life was not affected (65.9%, 96.5%, and 92.2% respectively) and only in the case of urinary discomfort did participants state that the impact was mild (28.6%) and moderate (5.5%; Fig. 3.

## Discussion

In this study carried out with a representative sample of women from the Tacna region, significant differences by age were found, being more frequent in young women (30–39 years). In comparison with other similar studies, Campbell reports 70% of dysfunction being in young women, distributed between UI and POP (70% and 18% respectively) [14] and in 2023 an interesting article was published about healthy women, professional dancers, where the presence of urinary leakage on exertion was 52.8% [15]; therefore, these findings can be considered relatively high in magnitude.

Age is also associated with a progressive increase in the prevalence of pelvic floor disorders, such as stress urinary incontinence (SUI), which increases with age and parity [16]. Consequently, the prevalence of BPD varies with age, showing a high incidence from an early age (30–39 years).

There is consistency with the results for age, parity, and number of births, as well as the effect of second and subsequent births [17, 18].

Other studies have shown that UI is more common in multiparous women than in nulliparous women, suggesting that the number of births and obstetric procedures may influence the occurrence of PFD and impact on women's quality of life [19–21].

In addition, pelvic floor muscle weakness, an important factor in PFD, may be related to pregnancy and childbirth, contributing to a higher prevalence of dysfunction in multiparous women, as reflected in this research.

Larsudd-Kåverud et al., in a recent publication evaluating POP with type of delivery and risk of future surgery, based on the Swedish National Quality Register of Gynecological Surgery, with more than 2 million registered women, found that those who had vaginal versus nulliparous or cesarean deliveries had a much higher risk of surgery for correction, which is corroborated in this research [22].

Parity, type of delivery, and interventions during delivery play a crucial role in the prevalence of PFD, with evidence suggesting a higher incidence in multiparous women, highlighting the importance of addressing this condition in the context of a woman's reproductive history.

These findings underline the need for careful assessment of women after childbirth, taking into consideration their obstetric history, to identify and effectively address PFDs.

In relation to studies of obesity and clinical symptomatology, De Oliveira et al. mentions significant differences between the diameter of the abdominal circumference with decreased pelvic floor muscle strength and symptoms of urinary leakage (*p* < 0.05) [23], Lai et al. argues that both central and general obesity are associated with UI, in agreement with the present study [24], mainly because of increased additional pressure on the pelvic floor.

These findings highlight the importance of addressing obesity as a significant modifiable risk factor when assessing the prevalence and management of PFD in overweight or obese women.

The relationship between PFD and physical exertion has been investigated in several studies. It has been observed that physical exercise can have both positive and negative effects on pelvic floor health. On the one hand, strengthening these muscles through specific exercises has been shown to be beneficial in the prevention and treatment of UI [25]. However, other studies suggest that certain types of high-impact exercise, such as weightlifting, may put additional pressure on the pelvic floor, which in turn may predispose to the development of PFD, especially in women who already have weakness in this area [26].

Physical exertion reported as high, medium, or low impact has been presented as an important risk factor for the presence of POP and UI. In this study, statistical impact was found only in those women with POP symptoms, but not in incontinent women, and this may be because urinary leakage is normalized or considered a taboo subject; thus, women do not report it, as mentioned by Elenskaia et al. [27]. For this reason, it is crucial to consider the type, intensity, and duration of exercise to assess the impact on pelvic floor health, and individualized assessment is important to determine the effect of physical exertion on PFD.

The relationship between caffeine and tobacco consumption and PFD is a topic of interest in medical research. Although no specific reference was found that directly addresses this relationship, previous studies have explored the effects of caffeine consumption on urogenital health. Specifically, caffeine consumption has been associated with bladder irritation and exacerbation of lower urinary tract symptoms in some individuals, with one scoping review indicating a decrease in urgency and bedwetting episodes with caffeine reduction, suggesting a potential link between caffeine and urinary symptoms [28].

Although some researchers provide evidence to support the association between caffeine consumption and exacerbation of lower urinary tract symptoms, the overall evidence is not entirely conclusive. Further research is needed to better understand the impact of caffeine on bladder function and exacerbation of urinary symptoms in certain individuals.

As for smoking, its impact on pelvic floor function has been investigated, especially in relation to the chronic cough associated with smoking, which may contribute to pelvic floor muscle weakness. There is evidence to support the impact of smoking on PFD, as highlighted by Lawson and Sacks, who noted that pre-pregnancy risk factors include pelvic floor muscle weakness, indicating a possible association between smoking and pelvic floor problems [8]. These references collectively caution that smoking may be a modifiable risk factor associated with PFD, particularly in the context of POP and SUI. As such, further research is needed to fully elucidate the specific mechanisms through which smoking contributes to weakness and PFD.

Finally, in relation to PFD and its impact on quality of life, it was found that UI is the disorder with highest self-reported mild and moderate discomfort, as opposed to colorectal or prolapse symptomatology. Despite the high prevalence, the discomfort caused by these pathological conditions is normalized by the general population, as reported by Fagerström Sade and López [29] and Hammad [30], in a systematic review, arguing that, from a medical perspective, the usual response is that these are normal, common, or incurable situations. Therefore, the identification of risk factors, together with adequate information and visibility of PFDs, is essential to initiate public health campaigns or interventions to improve the quality of life of these women.

This study is relevant because it is to our knowledge the first national study to reveal the high frequency of PFD and its impact on quality of life in the Tacna region. The findings will allow health service providers at governmental and health care levels to develop strategies for prevention, early diagnosis, and treatment of these pathological conditions.

The fact that this is a cross-sectional study is a limitation because causality cannot be established. On the other hand, the weight of the women was recorded by self-report and not by scale measurement. Additionally, we consider it important to expand the study with a larger number of women living in rural areas, as they exhibit different habits, physical characteristics, and educational levels. This would provide more evidence to effectively address the improvement of these dysfunctions, serving as a basis for public health initiatives in Peru. For future studies on this matter, we recommend developing a transcultural and comparative review of sociodemographic attributes and quality of life of women with PFD.

## Conclusion

Pelvic floor dysfunction in women is very common and it is strongly associated with overweight, obesity, parity, route of delivery, and physical exertion. The impact on quality of life was mild and moderate for those with urinary discomfort.

## Data Availability

We declare that the database is available upon request.
